# Fabrication of an Azithromycin Sensor

**DOI:** 10.3390/bios13110986

**Published:** 2023-11-16

**Authors:** Theophile Niyitanga, Mohd Quasim Khan, Khursheed Ahmad, Rais Ahmad Khan

**Affiliations:** 1 School of Materials Science and Engineering, Yeungnam University, Gyeongsan 38541, Republic of Korea; t.niyitanga@yu.ac.kr; 2 Department of Chemistry, M.M.D.C, Moradabad, M.J.P. Rohilkhand University, Bareilly 244001, UP, India; 3 Department of Chemistry, College of Science, King Saud University, Riyadh 11451, Saudi Arabia

**Keywords:** Ti_3_AlC_2_, MoS_2_, MAX, azithromycin, sensors

## Abstract

Azithromycin (AZY) is a well-known top-prioritized antibiotic and is used by humans in strong concentrations. However, the side effects of the AZY antibiotic may cause some serious and significant damage to humans and the environment. Thus, there is a need to develop effective and sensitive sensors to monitor accurate concentrations of AZY. In the last decade, electrochemistry-based sensors have received enormous attention from the scientific community because of their high sensitivity, selectivity, cost-effectiveness, fast response, rapid detection response, simple fabrication, and working principle. It is important to mention that electrochemical sensors rely on the properties of electrode modifiers. Hence, the selection of electrode materials is of great significance when designing and developing efficient and robust electrochemical sensors. In this study, we fabricated an AZY sensor by utilizing a molybdenum disulfide/titanium aluminum carbide (MoS_2_@Ti_3_AlC_2_) composite as the electrode material. The MoS_2_@Ti_3_AlC_2_ composite was synthesized via a simple sonication process. The synthesized MoS_2_@Ti_3_AlC_2_ composite was characterized using a powder X-ray diffraction (XRD) method to examine the phase purity and formation of the MoS_2_@Ti_3_AlC_2_ composite. Scanning electron microscopy (SEM) was used to study the surface morphological features of the prepared MoS_2_@Ti_3_AlC_2_ composite, whereas energy dispersive X-ray spectroscopy (EDAX) was adopted to determine the elemental composition of the prepared MoS_2_@Ti_3_AlC_2_ composite. The glassy carbon (GC) electrode was modified with the prepared MoS_2_@Ti_3_AlC_2_ composite and applied as the AZY sensor. The sensing performance of the MoS_2_@Ti_3_AlC_2_ composite-modified GC electrode was studied using linear sweep voltammetry. The sensor demonstrated excellent performance when determining AZY and showed a good detection limit of 0.009 µM with a sensitivity of 6.77 µA/µM.cm^2^.

## 1. Introduction

Antibiotics serve as commonly prescribed medications for the treatment of critically ill patients, aiming to impede the progression of bacterial infections characterized by variations in individual drug metabolism [1,2]. The assessment of antibiotic levels and non-invasive care involves examining biological fluids, such as through combination therapy or polytherapy [3]. This approach unveils the correlation between the antibiotic dosage and its impact by revealing the current status of treatment [4]. Azithromycin (AZY; N-methyl-9a-aza-9-deoxo-9-dihydro-9a-homoerythromycin A), is a member of the macrolide antibiotic family with 15-membered azalides. AZY is employed for the treatment of various bacterial infections [5,6]. AZY is primarily utilized in either mono or polytherapy to address both gram-positive and gram-negative bacterial infections, and it is notably applied in chronic inflammatory conditions like allergy, asthma, autoimmune diseases, coeliac disease, hepatitis, and inflammatory bowel diseases [7]. In AZY therapy, various unwanted effects and negative responses, such as diarrhea, nausea, and the discoloration of teeth, eyes, and skin, are documented [8]. When AZY is used in combination with simvastatin/atorvastatin as part of polytherapy, there is an elevated risk of rhabdomyolysis or an increased likelihood of cardiovascular death [9]. Additionally, the inadvertent presence of pharmaceutical compounds is prevalent in aquatic environments, mainly due to the widespread use of antibiotics in treating human and animal ailments [10,11]. Given that antibiotics, which are largely water-soluble, are extensively used and approximately 90% of the dosage is excreted through urine, these compounds find their way into wastewater systems without undergoing significant alterations, resulting in a diverse mixture of metabolites [10]. Thus, it is necessary to monitor the level of AZY [11]. 

Researchers have employed various spectroscopic, microbiological, and separation techniques to quantify the concentration of AZY [12,13]. While these methods offer commendable sensitivity and accuracy, they suffer from drawbacks, such as the need for sophisticated equipment, time-consuming analyses, and lengthy sample pretreatment processes [14]. To address these issues, electrochemical determination emerges as a superior alternative, characterized by a straightforward setup procedure, minimal resource usage, tunable selectivity, and high reproducibility and portability, rendering it a cost-effective analytical instrument [15,16,17,18,19]. The properties of electrode modifiers play a significant role in the sensing behavior of electrochemical sensors [18].

Recently, MoS_2_, also known as molybdenum disulfide, a type of two-dimensional transition metal dichalcogenide (TMD), has captured the attention of the scientific community due to its distinctive electronic, optical, and mechanical characteristics [20,21,22]. Its potential applications in diverse fields, including electronics, catalysis, energy storage, and lubrication, have fueled a growing interest in MoS_2_ [23]. Structurally, MoS_2_ consists of molybdenum atoms situated between two layers of sulfur atoms, forming a hexagonal lattice structure reminiscent of graphene [24]. Notably, MoS_2_ exhibits notable mechanical strength, impressive thermal conductivity, and chemical stability, rendering it an appealing material for applications in nanoelectronics and nanomechanical systems [25]. The exploration of MoS_2_ has paved the way for innovative research, and its unique properties continue to captivate the scientific community’s curiosity [26,27]. The hybrid composites of MoS_2_ have been widely used as electrode modifiers for various applications, including sensing, energy storage, and other optoelectronic applications [21,22,23].

In the past few years, materials possessing the MAX phase have been denoted as M_n+1_AX_n_, where M represents early transition metals, A stands for III A or IV A elements, and X could be nitrogen or carbon with a range of n from one to three [28]. These MAX materials typically exhibit a non-oxide composition with a highly layered hexagonal architecture, and they have recently garnered significant attention across various research domains [29]. Similar to metals, MAX materials demonstrated heightened electrical and thermal conductivity along with excellent chemical stability [30]. This investigation also revealed a high density and resistance to oxidation. Notably, titanium aluminum carbide (Ti_3_AlC_2_), belonging to the 2D layered ternary carbide family, exemplifies these characteristics. The structural configuration of Ti_3_AlC_2_ involves highly sequential arrangements of structural slabs within unit cells, contributing to superior electrical and mechanical properties [31,32]. Given these attributes, the 2D layers of Ti_3_AlC_2_ are considered an ideal platform for hosting nanoparticles in catalytic applications [33,34]. It was reported that the presence of synergistic interactions in hybrid composite materials may show better electrocatalytic properties [33]. In this context, a novel composite of Ti_3_AlC_2_@SmVO_4_ heterojunction was reported for the fabrication of sulfathiazole sensors [35]. This showed that Ti_3_AlC_2_-based composite materials would be a hot topic for the determination of antibiotics and other hazardous compounds [35]. 

In this study, we demonstrated the fabrication of a MoS_2_@Ti_3_AlC_2_ composite using a two-step synthetic procedure. Furthermore, the AZY sensor was developed by modifying the surface of a glassy carbon electrode via the MoS_2_@Ti_3_AlC_2_ composite as an electrode modifier. So far, no report is available on the use of a MoS_2_@Ti_3_AlC_2_ composite as an electrode modifier for the fabrication of an AZY sensor. This is the first report that demonstrates the role of a MoS_2_@Ti_3_AlC_2_ composite as an AZY sensing material. 

## 2. Experimental Section

### 2.1. Synthesis of MoS_2_@Ti_3_AlC_2_

In the first step, MoS_2_ was prepared by employing a hydrothermal synthetic procedure. The MoS_2_ was obtained via the hydrothermal treatment of a Mo and S precursor solution. Briefly, 420 mg of sodium molybdate Na_2_MoO_4_.2H_2_O was dissolved in 50 mL of deionized water. Following this, 620 mg of thiourea (NH_2_CSNH_2_) was introduced into the solution. The resulting blend was stirred for half an hour at room temperature. 

**Scheme 1:**
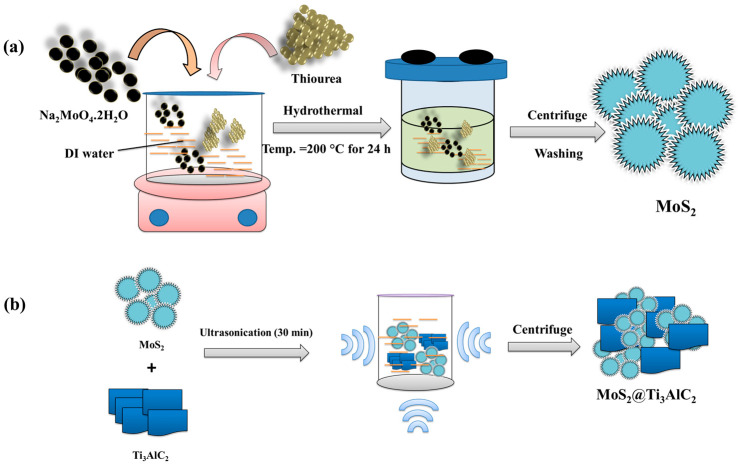
Schematic of the synthetic procedure for MoS_2_ (**a**) and MoS_2_@Ti_3_AlC_2_ (**b**).

Subsequently, the mixture was transferred to a steel autoclave and subjected to vacuum furnace conditions at 200 °C for a duration of 24 h (Scheme 1a). The resulting product underwent washing with deionized water and ethanol, followed by drying in a vacuum oven at 70 °C overnight. Furthermore, the MoS_2_@Ti_3_AlC_2_ composite was prepared via a simple sonochemical procedure. A total of 200 mg of Ti_3_AlC_2_ was mixed with 300 mg of the hydrothermally prepared MoS_2_ in 25 mL of deionized water (DI water). This aqueous reaction mixture was subjected to ultrasonication for 1 h to obtain the suspension of the MoS_2_@Ti_3_AlC_2_ composite (Scheme 1b). The final product was washed, centrifuged, and kept in a vacuum oven for drying at 70 °C. 

### 2.2. Apparatus

The present study involves the utilization of X-ray diffraction (XRD), scanning electron microscopy (SEM), and energy-dispersive X-ray spectroscopy (EDX) techniques to characterize the phase purity and the surface morphological and elemental composition of the prepared MoS_2_@Ti_3_AlC_2_ composite. The XRD patterns of the prepared samples were recorded on a RINT 2500 V powder X-ray diffractometer (Rigaku; Tokyo, Japan) with Cu/K_α irradiation (λ = 1.5406 Å). The SEM images of the prepared samples were collected on the Supra 55 Zeiss Oxford microscope. The Oxford Instruments X-max Aztec spectroscope was adopted for the recording of the EDX spectrum and EDX mapping images of the prepared samples. The CH instrument (three-electrode system) was used for electrochemical sensing studies. The counter electrode was a platinum wire-based electrode, whereas Ag/AgCl was used as a reference electrode. The GC electrode or modified GC electrodes were used as the working or sensing electrodes. 

### 2.3. Materials 

The materials, solvents, and initial components (sodium molybdate Na_2_MoO_4_.2H_2_O, ACS reagent, ≥99.0%; thiourea NH_2_CSNH_2_, ACS reagent, ≥99.0%; and azithromycin (AZY) used in this study were obtained from Merck. All the other chemical substances were utilized in their original state without undergoing additional purification processes.

### 2.4. Fabrication of the Sensor

**Scheme 2:**
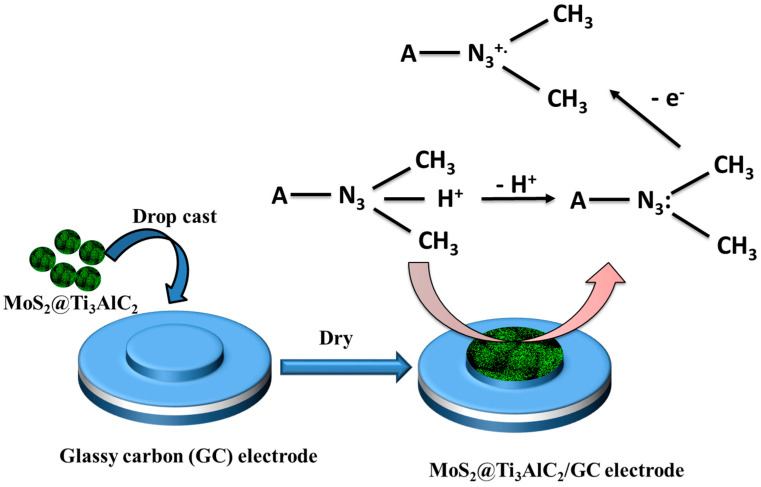
Schematic description of the surface modification of the GC electrode.

The active surface of the GC electrode was cleaned by using a 0.5 µm alumina slurry and pad. This was further cleaned by dipping it in ethanol solution for 60 s. Finally, the electrode-modifying materials (MoS_2_, Ti_3_AlC_2,_ and MoS_2_@Ti_3_AlC_2_) were dispersed in DI water. Next, 5 mg of the electrode-modifying material (MoS_2_, Ti_3_AlC_2_, or MoS_2_@Ti_3_AlC_2_) was added to 1 mL of DI water and sonicated for 30 min. Furthermore, 7 µL of the prepared aqueous solution was drop-casted on the GC electrode and dried in an oven at 40 °C. The GC modification is illustrated in Scheme 2. The bare GC electrode was used as GC-1. The GC electrode modified with MoS_2_, Ti_3_AlC_2_, and MoS_2_@Ti_3_AlC_2_ were used as MoS_2_/GC-3, Ti_3_AlC_2_/GC-2, and MoS_2_@Ti_3_AlC_2_/GC-4, respectively. 

## 3. Results and Discussion

### 3.1. Material Characterization

**Figure 1:**
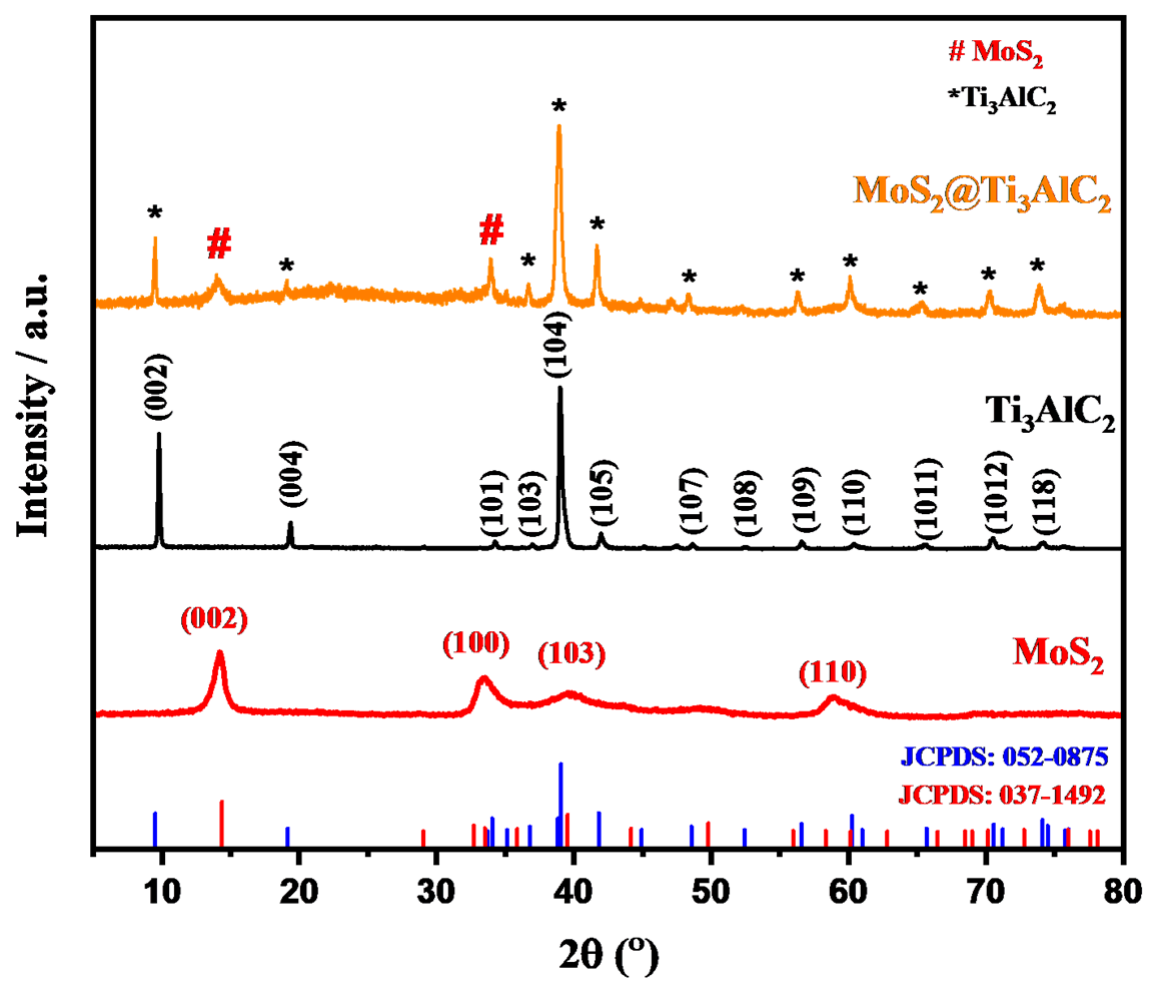
XRD patterns of the synthesized MoS_2_, Ti_3_AlC_2_, and MoS_2_@Ti_3_AlC_2_ composites.

The phase and crystalline nature of the purchased Ti_3_AlC_2_ and synthesized samples (MoS_2_ and MoS_2_@Ti_3_AlC_2_ composite) were characterized by recording the XRD patterns in the 2theta range of 5–80° at a scan rate of 2°/min. The recorded XRD patterns of the MoS_2_, Ti_3_AlC_2_, and MoS_2_@Ti_3_AlC_2_ composite are displayed in Figure 1. The XRD pattern of the MoS_2_ composite exhibited four major diffraction peaks at 14.11°, 33.40°, 40.12°, and 58.93° which suggested the presence of (002), (100), (103), and (110), diffraction planes, respectively. These results are well-matched with reported JCPDS data 037-1492. The XRD pattern of the Ti_3_AlC_2_ phase demonstrated the presence of various diffraction peaks at 9.80°, 19.29°, 34.22°, 36.97°, 38.89°, 41.85°, 48.62°, 52.46°, 56.59°, 60.35°, 65.58°, 70.44°, and 74.10°. 

The above-mentioned XRD peaks could be assigned to the (002), (004), (101), (103), (104), (105), (107), (108), (109), (110), (1011), (1012), and (118) diffraction planes. These results are well-matched with the published JCPDS data 052-0875. The XRD pattern of the synthesized MoS_2_@Ti_3_AlC_2_ composite showed (002), (004), (101), (103), (104), (105), (107), (108), (109), (110), (1011), (1012), and (118) diffraction planes for the presence of Ti_3_AlC_2_ and two other (002) and (100) diffraction planes for MoS_2_. This authenticated the formation of the MoS_2_@Ti_3_AlC_2_ composite. The high intensities indicated the presence of a decent crystalline nature of the prepared MoS_2_@Ti_3_AlC_2_ composite. 

**Figure 2:**
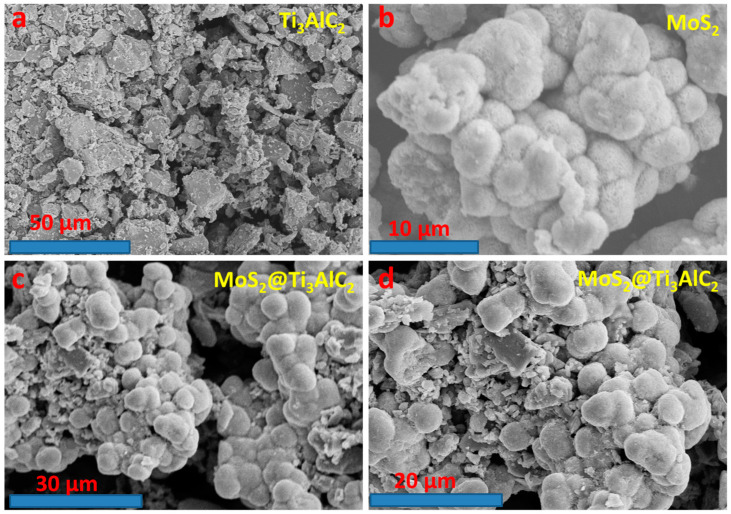
SEM image of the Ti_3_AlC_2_ (**a**), MoS_2_ (**b**), and MoS_2_@Ti_3_AlC_2_ composites (**c**,**d**).

The surface morphology of the samples (MoS_2_, Ti_3_AlC_2,_ and MoS_2_@Ti_3_AlC_2_ composites) were analyzed using SEM. Figure 2a exhibits the presence of Ti_3_AlC_2_ flake-like surface structures. The SEM results for the synthesized MoS_2_ are shown in Figure 2b. It can be seen that MoS_2_ demonstrated a flower-like surface morphology, and these flowers were agglomerated or interconnected to form a sheet-like surface. The SEM images for the synthesized MoS_2_@Ti_3_AlC_2_ composite were also recorded and are displayed in Figure 2c,d.

The SEM results for the synthesized MoS_2_@Ti_3_AlC_2_ composite showed the presence of MoS_2_ on the Ti_3_AlC_2_ surface. It can be observed from the SEM results that MoS_2_ was incorporated or embedded in the Ti_3_AlC_2_ surface. This suggested that the MoS_2_@Ti_3_AlC_2_ composite was successfully formed, as shown in Figure 2c,d.

**Figure 3:**
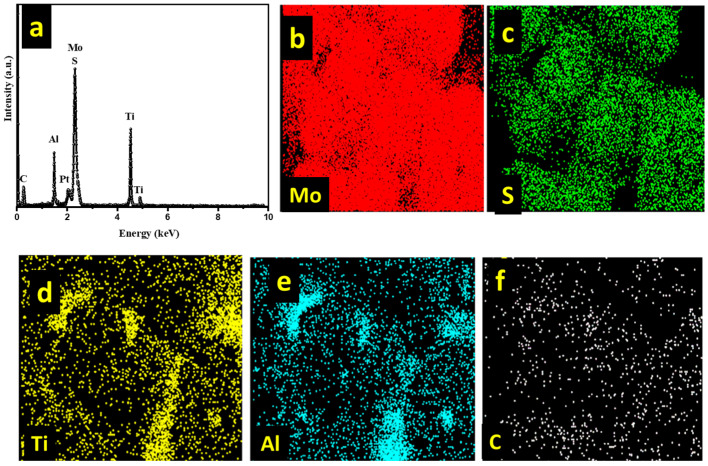
EDX spectrum of the synthesized MoS_2_@Ti_3_AlC_2_ composite (**a**) and the elemental mapping images for Mo (**b**), S (**c**), Ti (**d**), Al (**e**), and C (**f**).

The presence of any other impurity or the confirmation of the formation of the MoS_2_@Ti_3_AlC_2_ composite was further authenticated by employing EDX spectroscopy. The recorded EDX spectroscopic data for the synthesized MoS_2_@Ti_3_AlC_2_ composite is displayed in Figure 3a. The obtained results demonstrated the presence of EDX signals, which were related to the presence of C, Al, Mo, S, and Ti. No additional impurity was observed, which revealed the excellent phase purity of the synthesized MoS_2_@Ti_3_AlC_2_ composite. The presence of the C, Al, and Ti elements was related to the Ti_3_AlC_2_ composite, whereas the Mo and S elements were related to the MoS_2_ composite, as shown in Figure 3a. The presence of Pt in the EDX spectrum was due to the coating of the Pt layer on the prepared samples for the SEM and EDX investigations.

The elemental mapping images for the synthesized MoS_2_@Ti_3_AlC_2_ composite were also recorded. The EDX mapping images for the Mo, S, Ti, Al, and S elements are shown in Figure 3b–f, respectively. The obtained results demonstrated the uniform distribution of the particles. These observations suggested that the MoS_2_@Ti_3_AlC_2_ composite was synthesized with excellent phase purity and reasonable morphological characteristics. 

### 3.2. Electrochemical Sensing Performance

It is well-known that electrochemical impedance spectroscopy (EIS) is the most significant tool to characterize modified GC electrodes. EIS also demonstrates and verifies the electrical conductivity and surface modification of GC electrodes. Electrode charge transfer resistance (Rct) plays a significant role in electrochemical sensing applications and depends on various factors, such as the dielectric or insulating parameters of the electrode and/or electrolyte interface. Rct can be determined using the EIS technique. Thus, we performed an EIS study to assess the electrocatalytic and charge transfer capabilities of the GC-1, Ti_3_AlC_2_/GC-2, MoS_2_/GC-3, and MoS_2_@Ti_3_AlC_2_/GC-4 composites. The EIS studies for the GC-1, Ti_3_AlC_2_/GC-2, MoS_2_/GC-3, and MoS_2_@Ti_3_AlC_2_/GC-4 composites were carried out in 5 mM [Fe(CN_6_)]^3−/4−^ in a 0.1 M KCl solution (Frequency range = 0.1–100 KHz). The obtained EIS results for the GC-1, Ti_3_AlC_2_/GC-2, MoS_2_/GC-3 and MoS_2_@Ti_3_AlC_2_/GC-4 composites are summarized in Figure S1a. It can be seen that GC-1 has a larger semi-circle with an Rct value of 1113.2 Ω, which suggests its poor conductive nature and low electrocatalytic properties. Ti_3_AlC_2_/GC-2 exhibits a relatively low semi-circle with an Rct value of 812 Ω. The EIS results for MoS_2_/GC-3 showed an Rct value of 571 Ω, whereas MoS_2_@Ti_3_AlC_2_/GC-4 had an Rct value of 379 Ω. Thus, it could be said that the electrical conductivity of the GC-1, Ti_3_AlC_2_/GC-2, MoS_2_/GC-3, and MoS_2_@Ti_3_AlC_2_/GC-4 composites follow the following trend below:

GC-1(Rct) **>** Ti_3_AlC_2_/GC-2(Rct) **>** MoS_2_/GC-3 (Rct) **>** MoS_2_@Ti_3_AlC_2_/GC-4 (Rct) (1)

Thus, MoS_2_@Ti_3_AlC_2_/GC-4 has high conductivity compared to the GC-1, Ti_3_AlC_2_/GC-2, and MoS_2_/GC-3 composites, as revealed by the EIS results. The cyclic voltametric (CV) responses of the GC-1, Ti_3_AlC_2_/GC-2, MoS_2_/GC-3, and MoS_2_@Ti_3_AlC_2_/GC-4 composites were also obtained in 5 mM [Fe(CN_6_)]^3−/4−^ in a 0.1 M KCl solution. Figure S1b demonstrates the obtained CV responses of the GC-1, Ti_3_AlC_2_/GC-2, MoS_2_/GC-3, and MoS_2_@Ti_3_AlC_2_/GC-4 composites. It is clear that the GC-1-based CV response has a poor electrocatalytic current response, whereas an improved current response was observed for Ti_3_AlC_2_/GC-2 and MoS_2_/GC-3. The highest current response was observed for MoS_2_@Ti_3_AlC_2_/GC-4. This suggested that MoS_2_@Ti_3_AlC_2_/GC-4 has good electrocatalytic properties and electrical conductivity. The above CV responses are consistent with the EIS results.

Linear sweep voltammetry (LSV) technology has received enormous attention due to its fast response and simplicity for the determination of various analytes. Thus, we adopted LSV technology as a sensing platform for the determination of AZY.

**Figure 4:**
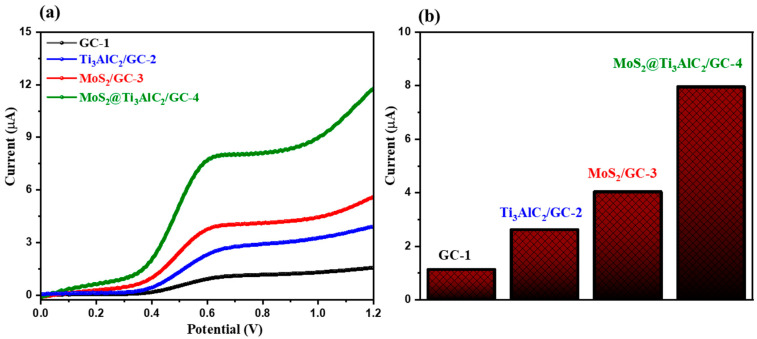
LSVs (**a**) and current responses (**b**) of GC-1, Ti_3_AlC_2_/GC-2, MoS_2_/GC-3, and MoS_2_@Ti_3_AlC_2_/GC-4 in 16 µM AZY in 0.1 M PBS (pH = 7) at a scan rate of 50 mV/s.

The LSV response of the GC-1, Ti_3_AlC_2_/GC-2, MoS_2_/GC-3, and MoS_2_@Ti_3_AlC_2_/GC-4 composites were studied in the presence of 16 µM AZY in 0.1 M PBS (pH = 7) at a scan rate of 50 mV/s. The obtained results are summarized in Figure 4a,b. GC-1 showed the lowest current response as compared to the other three modified electrodes (Ti_3_AlC_2_/GC-2, MoS_2_/GC-3, and MoS_2_@Ti_3_AlC_2_/GC-4). The highest current response was observed for MoS_2_@Ti_3_AlC_2_/GC-4 for the determination of AZY.

MoS_2_ catalyzes the oxidation of AZY, whereas the presence of Ti_3_AlC_2_ (acting as conductive support) may enhance the electron transport rate. Thus, an improved current response was observed for MoS_2_@Ti_3_AlC_2_/GC-4 towards the oxidation of AZY. Therefore, it could be understood that the presence of synergistic interactions, such as the excellent catalytic properties of MoS_2_ and the high conductivity of the MAX phase (Ti_3_AlC_2_), may improve the electrocatalytic behavior of MoS_2_@Ti_3_AlC_2_/GC-4, which resulted in the enhanced current response. Thus, it would be worth using MoS_2_@Ti_3_AlC_2_/GC-4 as a sensing electrode for further studies, such as the effect of concentration, selectivity, stability etc. Hence, we used MoS_2_@Ti_3_AlC_2_/GC-4 as the final working electrode for further studies. The effect of mass loading (the MoS_2_@Ti_3_AlC_2_ amount on the GC surface) was also optimized. The observations showed that the GC electrode coated with 7 µL of MoS_2_@Ti_3_AlC_2_ is more efficient for the determination of AZY (Figure S2).

The pH of the solution may also have a significant role in the determination of AZY. Thus, the pH of the solution was also optimized. The results show the highest performance of MoS_2_@Ti_3_AlC_2_/GC-4 at a pH value of 7.0 (Figure S3). The LSV response of MoS_2_@Ti_3_AlC_2_/GC-4 in the absence of AZY was also recorded in 0.1 M PBS at a scan rate of 50 mV/s. The obtained results are provided in Figure S4. It can be seen that no oxidation peak was observed in the absence of AZY.

**Figure 5:**
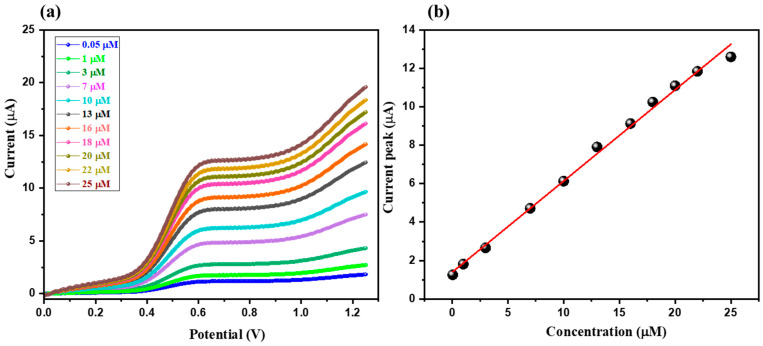
LSV responses (**a**) and calibration curves (**b**) of MoS_2_@Ti_3_AlC_2_/GC-4 in different concentration of AZY (0.05 1, 3, 7, 10, 13, 16, 18, 20, 22, and 25 µM) in 0.1 M PBS (pH = 7.0) at a scan rate of 50 mV/s.

The concentration of AZY may also have a great impact on the current response of MoS_2_@Ti_3_AlC_2_/GC-4. Thus, time is needed to examine the effect of various concentrations of AZY on the current response of MoS_2_@Ti_3_AlC_2_/GC-4. The LSV responses of MoS_2_@Ti_3_AlC_2_/GC-4 in different concentrations of AZY (0.05 1, 3, 7, 10, 13, 16, 18, 20, 22, and 25 µM) in 0.1 M PBS (pH = 7.0) at a scan rate of 50 mV/s were obtained. The obtained results are summarized in Figure 5a,b. It can be noted that the current response increased with an increase in AZY concentration, as shown in Figure 5a. The calibration plot was drawn between the peak current responses versus the concentrations of AZY in Figure 5b. The calibration curve suggests that the current response for the determination of AZY linearly increases with R^2^ = 0.99.

**Figure 6:**
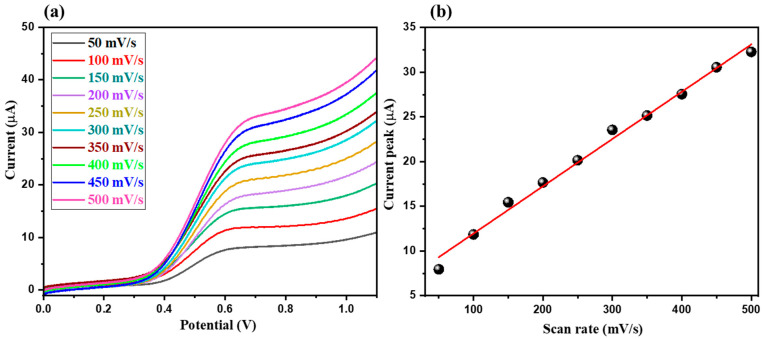
LSV responses (**a**) of MoS_2_@Ti_3_AlC_2_/GC-4 in 16 µM AZY in 0.1 M PBS (pH = 7.0) at various scan rates of 50–500 mV/s. Corresponding calibration curve (**b**) of the current versus applied scan rates.

The effect of the various applied scan rates were by recording LSV graphs of MoS_2_@Ti_3_AlC_2_/GC-4 in the presence of 16 µM AZY in 0.1 M PBS (pH = 7.0) at various scan rates of 50–500 mV/s. The recorded LSV graphs of MoS_2_@Ti_3_AlC_2_/GC-4 in the presence of 16 µM AZY in 0.1 M PBS (pH = 7.0) at various scan rates of 50–500 mV/s are summarized in Figure 6a. It was noted that the current response of MoS_2_@Ti_3_AlC_2_/GC-4 increases with respect to the applied scan rate. The calibration curve between the peak current responses against the applied scan rate is depicted in Figure 6b. The current response linearly increases, as suggested by the linear calibration curve between the peak current responses versus the applied scan rates (Figure 6b). The calibration plot showed an R^2^ value of 0.99. Therefore, it could be assumed that the determination of AZY is an adsorption-controlled process.

**Figure 7:**
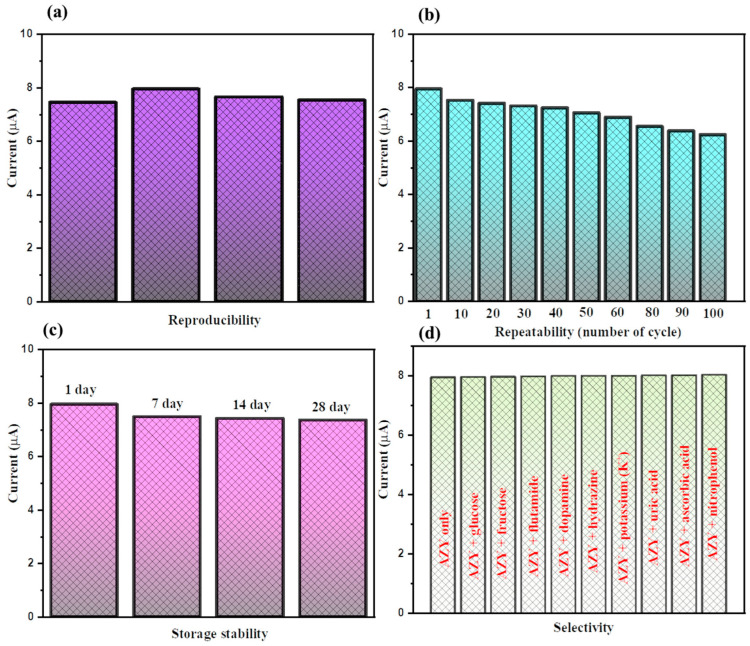
The reproducibility (**a**), repeatability (**b**), storage stability (**c**), and selectivity (**d**) of MoS_2_@Ti_3_AlC_2_/GC-4 in 16 µM AZY in 0.1 M PBS (pH = 7.0) at a scan rate of 50 mV/s.

The reproducibility, repeatability, selectivity, and storage stability of the electrochemical sensors are the most desirable characteristics for their potential applications. In this context, four GC electrodes were fabricated under similar conditions. The current response of the four freshly fabricated GC electrodes (with MoS_2_@Ti_3_AlC_2_ as the electrode material) in the presence of 16 µM AZY was assessed. The obtained current responses are summarized in Figure 7a. The reproducibility study showed reasonable performance and suggested the presence of good reproducible features in the fabricated electrodes. Similarly, we assessed the repeatability of MoS_2_@Ti_3_AlC_2_/GC-4 in 16 µM AZY and obtained the current responses for different cycles, which are depicted in Figure 7b. The observations showed that MoS_2_@Ti_3_AlC_2_/GC-4 retained more than 85% of the current response compared to the initial current response after 100 cycles. Thus, it could be understood that MoS_2_@Ti_3_AlC_2_/GC-4 has good repeatability for the determination of AZY. MoS_2_@Ti_3_AlC_2_/GC-4 was stored for various days, and its current response was assessed. According to Figure 7c, it can be seen that MoS_2_@Ti_3_AlC_2_/GC-4 has good storage stability for up to 28 days.

The selectivity of MoS_2_@Ti_3_AlC_2_/GC-4 is one of the most significant features for its practical application. In this regard, the effect of interfering compounds on the current response of MoS_2_@Ti_3_AlC_2_/GC-4 was examined. The concentration of AZY was 16 µM, whereas the concentration of the interfering compound was five times higher than that of AZY. Glucose, fructose, flutamide, dopamine, hydrazine, potassium, uric acid, ascorbic acid, and nitrophenol were used as interfering species. The obtained responses for the current responses are summarized in Figure 7d. These results suggest that an insignificant change in the current response of MoS_2_@Ti_3_AlC_2_/GC-4 was observed. The LSV responses of MoS_2_@Ti_3_AlC_2_/GC-4 in the 16 µM AZY and 16 µM AZY + 80 µM interferences (glucose, fructose, flutamide, dopamine, hydrazine, potassium, uric acid, ascorbic acid, and nitrophenol) in 0.1 M PBS (pH = 7.0; scan rate = 50 mV/s) are presented in Figure S5. No other compound was detected in the LSV response, as shown in Figure S5. Thus, it is clear that MoS_2_@Ti_3_AlC_2_/GC-4 possesses good selective properties for the determination of AZY. The probable mechanism for the determination of AZY is illustrated in Scheme 2.

The performance of MoS_2_@Ti_3_AlC_2_/GC-4 was evaluated by calculating its limit of detection, i.e., LOD, and sensitivity for the detection of AZY. The following equations were used for the calculation of the LOD and the sensitivity of MoS_2_@Ti_3_AlC_2_/GC-4 for the determination of AZY [36].

LOD = 3 × σ/slope (2)

(Herein, σ = standard error)

Sensitivity = Slope/Area (3)

The calculated LOD and sensitivity of MoS_2_@Ti_3_AlC_2_/GC-4 are summarized in Table 1 with the previously reported articles. In the previous studies, molecularly imprinted polymer (MIP)/acetylene black was used as the electrode material and showed a LOD of 0.07 µM [35]. Another report also exhibited the potential of a MIP/carbon paste electrode (CPE) for the determination of AZY [36]. Other electrode materials, such as gold nano-urchin/graphene oxide (GO) [37], MIP/2,2‘-bithiophene/3-thienyl boronic acid [38], and GO/multi-walled carbon nanotubes (MWCNT) [39] were reported for the detection of AZY. Our obtained results are reasonable in terms of the LOD, as shown in Table 1. In the previous studies, the determination of AZY involved an electro-oxidation process, as listed in Table 1. The MoS_2_@Ti_3_AlC_2_-modified GC electrode also demonstrated an electro-oxidation process for the determination of AZY. The obtained results in this study are consistent with previous studies [35,36,37,38,39].

## 4. Conclusions

To summarize, a novel composite of MoS_2_ and Ti_3_AlC_2_ was synthesized using a hydrothermal-assisted sonochemical method. The obtained MoS_2_@Ti_3_AlC_2_ composite was characterized using sophisticated techniques, which confirmed the formation of the MoS_2_@Ti_3_AlC_2_ composite with good phase purity. The obtained MoS_2_@Ti_3_AlC_2_ composite was further used as an AZY sensing material. The GC surface was modified with the MoS_2_@Ti_3_AlC_2_ composite via a drop-cast approach. The MoS_2_@Ti_3_AlC_2_ composite-modified GC electrode exhibited excellent performance. The reasonable detection limit of 0.009 µM with a sensitivity of 6.77 µA/µM.cm^2^ were achieved using the LSV method. The MoS_2_@Ti_3_AlC_2_ composite-modified GC electrode imposed remarkably good results, such as selectivity, stability, and sensitivity. This straightforward identification of AZY has the potential to unlock numerous opportunities in the pharmaceutical and industrial fields, thanks to its minimal constraints.

## Figures and Tables

**Table 1:** Comparison of the sensing performance with previous studies.

Material	Techniques	LOD (µM)	Sensitivity (µA/µM.cm^2^)	Linear Range (µM)	Detection Process	References
MoS_2_@Ti_3_AlC_2_	LSV	0.009	6.77	0.05–25	oxidation	This study
MIP/acetylene black	DPV	0.07	-	0.1–20	oxidation	[35]
MIP/CPE	electrochemiluminescence	2.3	-	1–100	oxidation	[36]
gold nano-urchin/GO	DPV	0.1	-	0.3–920	oxidation	[37]
MIP/2,2‘-bithiophene/3-thienyl boronic acid	EIS	0.85	-	13.33–66.67	oxidation	[38]
GO/MWCNT	LSV	0.07	-	0.1–10	oxidation	[39]

## Data Availability

Data are contained within the article and Supplementary Materials.

## Supplementary Materials

The following supporting information can be downloaded at: https://www.mdpi.com/article/10.3390/bios13110986/s1, Figure S1: Nyquist plots (a) and CVs (b) of the GC-1, Ti_3_AlC_2_/GC-2, MoS_2_/GC-3 and MoS_2_@Ti_3_AlC_2_/GC-4 in 5 mM [Fe(CN_6_)]^3-/4-^ in 0.1 M KCl solution (Frequency range = 0.1-100 KHz for EIS graph). Scan rate = 50 mV/s (for CV graph).; Figure S2: Current response of the MoS_2_@Ti_3_AlC_2_/GC-4 (different mass loading; 3, 5, 7 and 9 µL) in 15 µM AZY in 0.1 M PBS (pH = 7) at scan rate of 50 mV/s.; Figure S3: Current response of the MoS_2_@Ti_3_AlC_2_/GC-4 (in 15 µM AZY in 0.1 M PBS (different pH; 1, 3, 5, 7, 9 and 11) at scan rate of 50 mV/s.; Figure S4: LSVs of the MoS_2_@Ti_3_AlC_2_/GC-4 in absence and presence of 16 µM AZY in 0.1 M PBS (pH = 7) at scan rate of 50 mV/s.; Figure S5: Selectivity: LSV response of the MoS_2_@Ti_3_AlC_2_/GC-4 in 16 µM AZY and 16 µM AZY + 80 µM interferences (glucose, fructose, flutamide, dopamine, hydrazine, potassium, uric acid, ascorbic acid, and nitrophenol) in 0.1 M PBS (pH = 7.0) at scan rates of 50 mV/s.
