# Supplementary material for: Fabrication of an Azithromycin Sensor
Source: Biosensors (Basel). 2023 Nov 16;13(11):986. doi: 10.3390/bios13110986 (PMC10669414; doi:10.3390/bios13110986)
Supplement: Supplementary file 1 [file biosensors-13-00986-s001.zip › biosensors-2700105-supplementary.pdf]

Supporting information

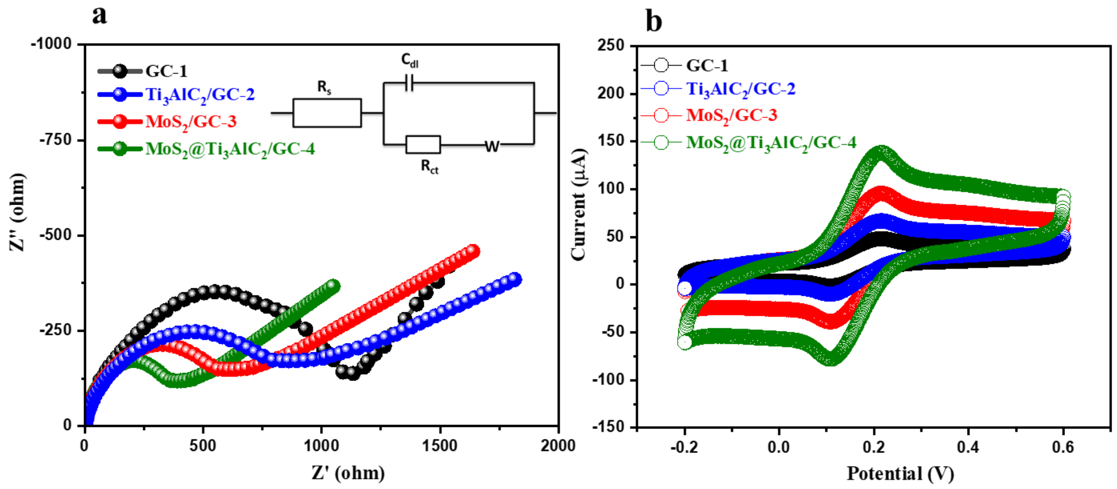

Figure S1. Nyquist plots (a) and CVs (b) of the GC-1, Ti_3AlC_2/GC-2, MoS_2/GC-3 and MoS_2@Ti_3AlC_2/GC-4 in 5 mM $[Fe(CN_6)]^{3-/4-}$ in 0.1 M KCl solution (Frequency range = 0.1-100 KHz for EIS graph). Scan rate = 50 mV/s (for CV graph).

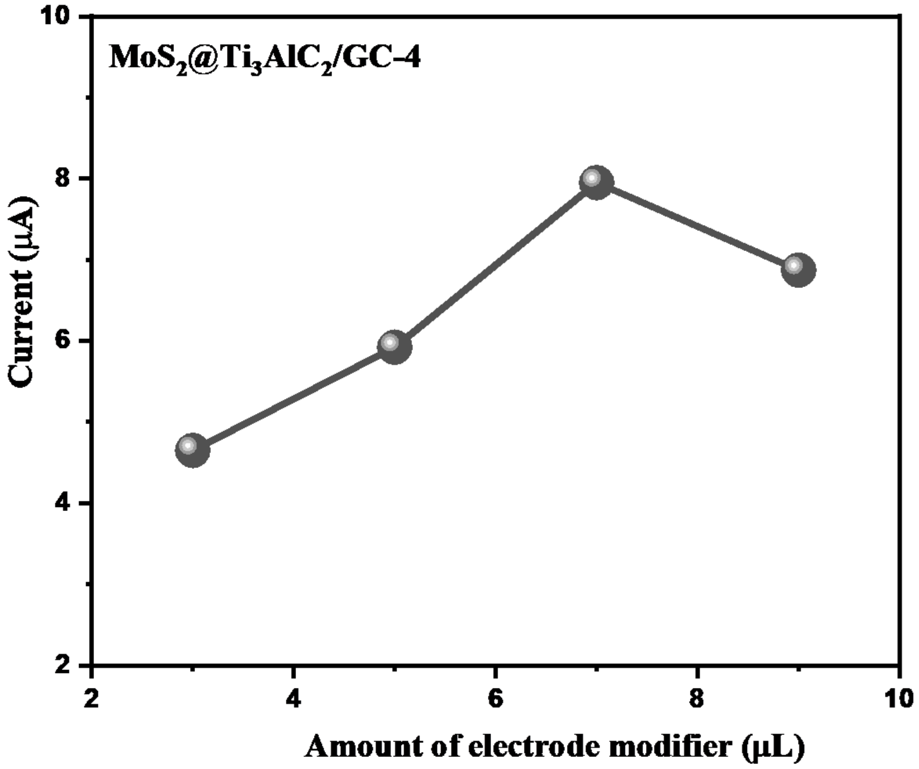

Figure S2. Current response of the $MoS_2@Ti_3AlC_2$/GC-4 (different mass loading; 3, 5, 7 and 9 µL) in 15 µM AZY in 0.1 M PBS (pH = 7) at scan rate of 50 mV/s.

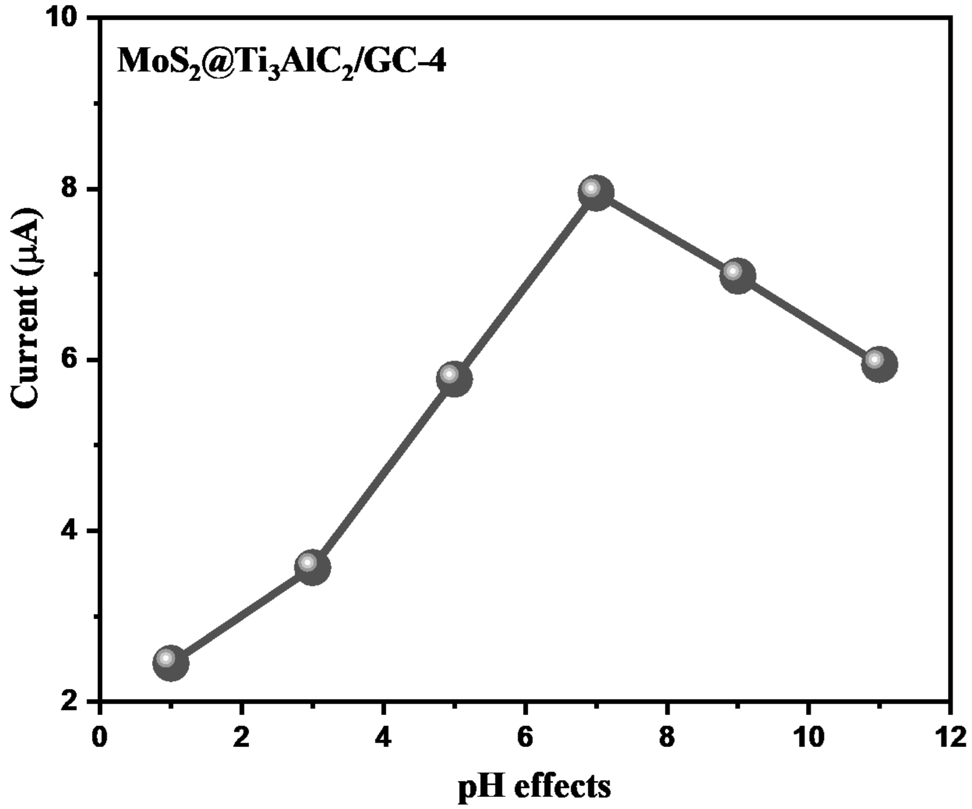

Figure S3. Current response of the $MoS_2@Ti_3AlC_2$/GC-4 (in 15 μM AZY in 0.1 M PBS (different pH; 1, 3, 5, 7, 9 and 11) at scan rate of 50 mV/s.

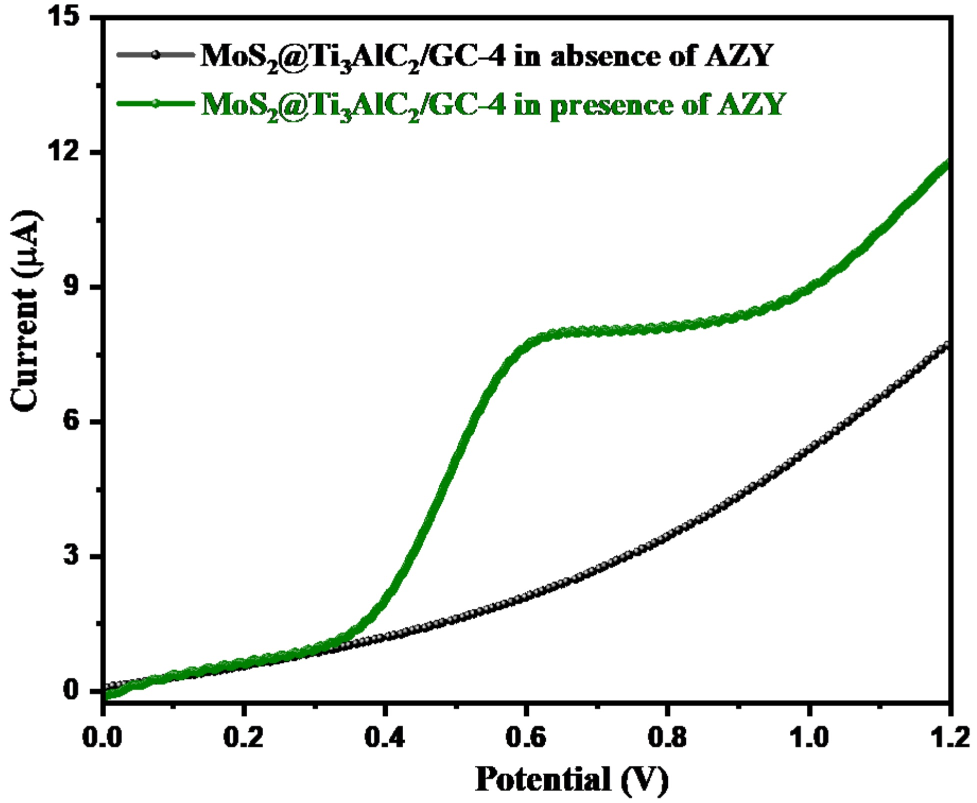

Figure S4. LSVs of the MoS_2@Ti_3AlC_2/GC-4 in absence and presence of 16 µM AZY in 0.1 M PBS (pH = 7) at scan rate of 50 mV/s.

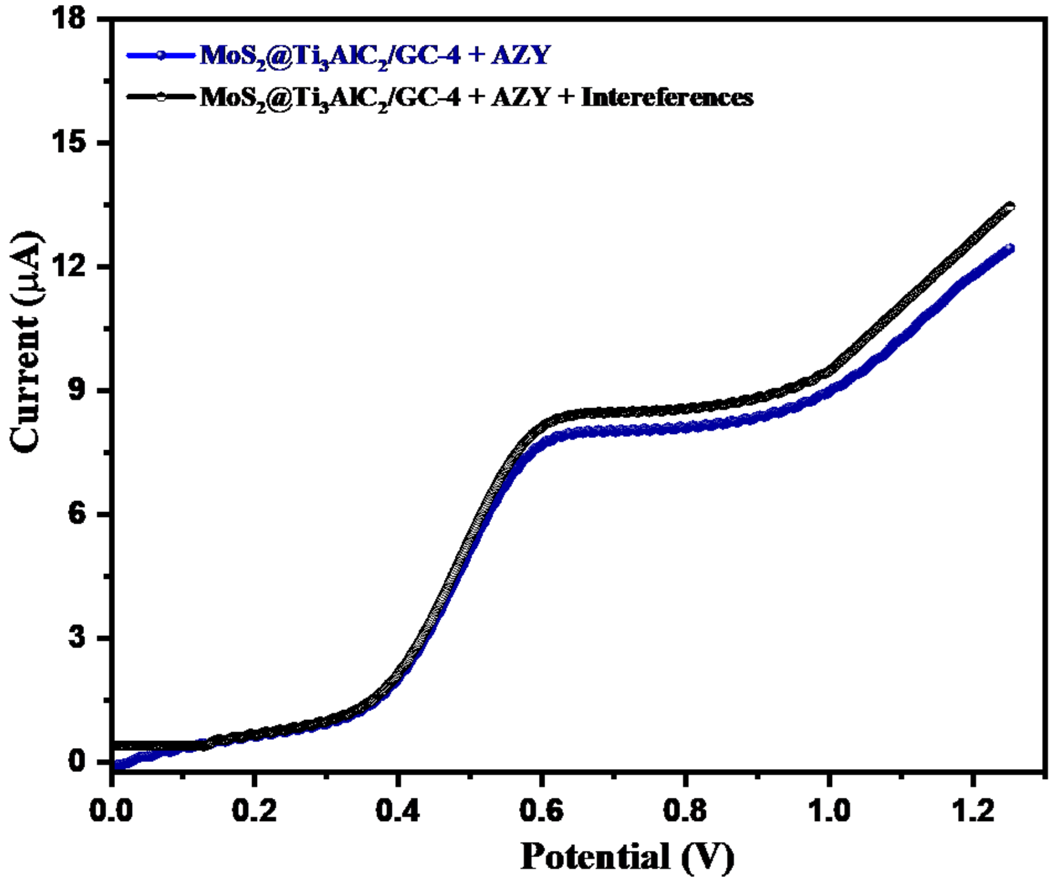

Figure S5. Selectivity: LSV response of the $MoS_2@Ti_3AlC_2$/GC-4 in 16 µM AZY and 16 µM AZY + 80 µM interferences (glucose, fructose, flutamide, dopamine, hydrazine, potassium, uric acid, ascorbic acid, and nitrophenol) in 0.1 M PBS (pH = 7.0) at scan rates of 50 mV/s.
